# Evaluation of SNOMED CT Grouper Accuracy and Coverage in Organizing the Electronic Health Record Problem List by Clinical System: Observational Study

**DOI:** 10.2196/51274

**Published:** 2024-05-09

**Authors:** Rashaud Senior, Timothy Tsai, William Ratliff, Lisa Nadler, Suresh Balu, Elizabeth Malcolm, Eugenia McPeek Hinz

**Affiliations:** 1 Duke University Health System, Durham, NC, United States; 2 Division of Primary Care and Population Health, Department of Medicine, Stanford University School of Medicine, Stanford, CA, United States; 3 Duke Institute for Health Innovation, Durham, NC, United States; 4 Division of General Internal Medicine, Duke University School of Medicine, Durham, NC, United States

**Keywords:** electronic health record, problem List, problem list organization, problem list management, SNOMED CT, SNOMED CT Groupers, Systematized Nomenclature of Medicine, clinical term, ICD-10, International Classification of Diseases

## Abstract

**Background:**

The problem list (PL) is a repository of diagnoses for patients’ medical conditions and health-related issues. Unfortunately, over time, our PLs have become overloaded with duplications, conflicting entries, and no-longer-valid diagnoses. The lack of a standardized structure for review adds to the challenges of clinical use. Previously, our default electronic health record (EHR) organized the PL primarily via alphabetization, with other options available, for example, organization by clinical systems or priority settings. The system’s PL was built with limited groupers, resulting in many diagnoses that were inconsistent with the expected clinical systems or not associated with any clinical systems at all. As a consequence of these limited EHR configuration options, our PL organization has poorly supported clinical use over time, particularly as the number of diagnoses on the PL has increased.

**Objective:**

We aimed to measure the accuracy of sorting PL diagnoses into PL system groupers based on Systematized Nomenclature of Medicine Clinical Terms (SNOMED CT) concept groupers implemented in our EHR.

**Methods:**

We transformed and developed 21 system- or condition-based groupers, using 1211 SNOMED CT hierarchal concepts refined with Boolean logic, to reorganize the PL in our EHR. To evaluate the clinical utility of our new groupers, we extracted all diagnoses on the PLs from a convenience sample of 50 patients with 3 or more encounters in the previous year. To provide a spectrum of clinical diagnoses, we included patients from all ages and divided them by sex in a deidentified format. Two physicians independently determined whether each diagnosis was correctly attributed to the expected clinical system grouper. Discrepancies were discussed, and if no consensus was reached, they were adjudicated by a third physician. Descriptive statistics and Cohen κ statistics for interrater reliability were calculated.

**Results:**

Our 50-patient sample had a total of 869 diagnoses (range 4-59; median 12, IQR 9-24). The reviewers initially agreed on 821 system attributions. Of the remaining 48 items, 16 required adjudication with the tie-breaking third physician. The calculated κ statistic was 0.7. The PL groupers appropriately associated diagnoses to the expected clinical system with a sensitivity of 97.6%, a specificity of 58.7%, a positive predictive value of 96.8%, and an *F*_1_-score of 0.972.

**Conclusions:**

We found that PL organization by clinical specialty or condition using SNOMED CT concept groupers accurately reflects clinical systems. Our system groupers were subsequently adopted by our vendor EHR in their foundation system for PL organization.

## Introduction

The electronic health record (EHR) problem list (PL) is a dynamic repository of a patient’s current and historical conditions as well as other health-related issues. As such, it supports communication across a wide range of potential caregivers and clinical environments. An accurate PL serves as a foundation for clinical care and population health management, with multiple derivative secondary processes, including phenotype extraction and disease prediction.

Understanding the history of the PL helps to illustrate why this construct has become the default format for summarizing patients’ clinical history. In the 1960s, Lawrence L Weed, MD, proposed the concepts of the problem-oriented medical record; the PL; and the Subjective, Objective, Assessment, and Plan (SOAP) notes for documentation [1]. The idea was to colocate clinical problems with clinical results to focus on systematically addressing all of a patient’s diagnoses [2]. Although the SOAP note became the standard format for clinical notes, the PL has encountered more inconsistent use, struggling with problems of inaccuracy, missing diagnoses, not being updated, and bloating [3]. In 2009, the HITECH (Health Information Technology Economic and Clinical Health) Act codified the requirement for an up-to-date PL for meaningful use [4]. Until recently, our vendor EHR had relied on relatively ineffective organization strategies for the PL.

**Figure 1.**
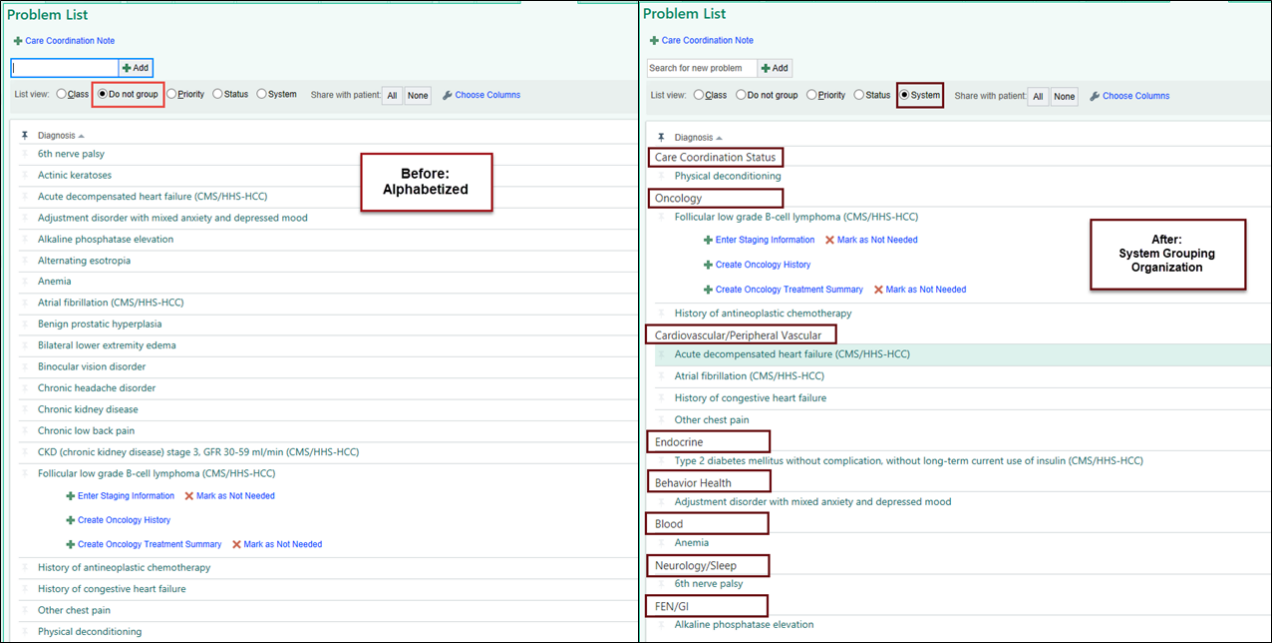
Appearance of a problem list before and after grouping algorithm application. Items were reorganized into 21 system groupers using Boolean logic with the Systematized Nomenclature of Medicine Clinical Terms (SNOMED CT) codes; they were then translated into the *International Classification of Diseases, Tenth Version* (*ICD-10*) codes. Groupers were based on a combination of traditional medical specialty categories, clinically relevant care coordination, and procedure-based groupings, some of which were themselves combined due to overlapping diagnostic coverage. The final order of the problem list items was determined by Epic System’s base hierarchy. CMS: The Centers for Medicare and Medicaid Services; HCC: Hierarchical Condition Category; HHS: US Department of Health and Human Services; FEN/GI: Fluids, Electrolytes, Nutrition/Gastrointestinal; GFR=Glomerular Filtration Rate.

With no one owner, the PLs have become disorganized and cluttered with duplications, conflicting entries, and no-longer-valid diagnoses that contribute to information overload and bloat, obscuring the patient’s clinical picture [5]. In its former state, our EHR PL was organized primarily alphabetically, with other options based on primary specialty or priority, all of which have limited clinical utility, especially as the number of diagnoses on the PL increases (Figure 1) . For example, for one patient, we found active diagnoses of lung nodule (Respiratory System), then lung cancer (Oncology System), and then lung cancer with brain metastases (Oncology System). These diagnoses were all related to the same problem but were added sequentially with previous diagnoses that were no longer clinically relevant and were not removed.

There are several major terminology standards that capture patient diagnoses, symptoms, and other health-related conditions, two of which are the *International Classification of Diseases* (*ICD*) [6] and the Systematized Nomenclature of Medicine Clinical Terms (SNOMED CT) [7]. The World Health Organization maintains *ICD* codes, which are designed to classify diseases, conditions, and other health-related issues [8,9]. Organized into 21 chapters, they use an alphanumeric classification format to identify diseases, injuries, and factors influencing health. The United States uses an additional Clinical Modifier for further specificity [10]. The *ICD* codes are used in various clinical and nonclinical settings, including disease description, treatment selection, billing, and research applications [11]. There are 78,044 total codes in the 2024 code set, according to the Centers for Medicare and Medicaid Services [12].

Currently managed by Systematized Nomenclature of Medicine (SNOMED) International (previously known as the International Health Terminology Standards Development Organization [IHTSDO]), SNOMED CT was designed to be a US standard for health information exchange. It functions as a highly granular ontology used to describe clinical observations and findings [13]. SNOMED CT uses a polyhierarchal (parent-child) format organized around a general root concept (eg, a clinical finding, procedure, situation with explicit context, or event) with increased granularity achieved by differentiating more specific descriptions of that root concept. This process allows for the representation of specific clinical content in a machine-readable format [14,15]. Updated monthly, the total number of concepts is 512,087 (as of April 1, 2024 [16]) and continues to increase over time.

Though required for billing, the *ICD, Tenth Revision* (*ICD-10)* terminology is not used directly in clinical care, as many code names are not consistent with clinical vernacular. For example, code Z91.038 “Allergy status to unspecified drugs, medicaments and biological substances” is not as intuitive as “Allergy to insect stings.” For this example, our third-party vendor, Intelligent Medical Objects (IMO), transforms the *ICD-10* codes into clinically relevant human-readable concepts. IMO additionally maps at least 1 SNOMED CT concept for each diagnosis, attached as metadata, upon which the PL groupers can be organized. Although SNOMED CT concepts are mapped to *ICD-10* codes [17,18], as with most ontologies, there are gaps in clinical concept coverage.

PL organization and cleanup is challenging for many reasons, including there being no single owner of a patient’s PL [19] and its maintenance and cleanup being secondary to other direct clinical care priorities. The tools in the EHR for cleanup are limited to a single patient and do not allow for automated processing or opportunities to categorize or define the state of the PL or large-scale maintenance at the population level. Multiple interventions, including reconfiguration of the EHR PL and re-education, have been met with limited success [20]. Despite these attempts, PL bloat and inaccuracy are widely recognized as issues affecting clinical care and secondary downstream uses of the data [3]. We have come to recognize that curating a clinically relevant and updated PL is a difficult challenge; our primary option for improving its organization was to extend and improve SNOMED CT groupers.

In this paper, we present a PL reorganization developed around clinical specialty groupings using SNOMED CT codes and Boolean logic. We describe the evaluation of the new PL groupers for clinical accuracy and efficiency using a convenience set of patients and their diagnoses. This system allows for future characterization of the PLs at the patient and population levels; it also provides potential for automated cleanup options in the future.

## Methods

### SNOMED CT Grouper Development and Evaluation

Author EMH extended and extensively modified 19 previously defined groupers initially developed by Heidi Twedt, MD, and added 2 newly defined system- or condition-based groupers, one for pediatric and one for transplant-specific conditions (Table 1). System groupers included traditional medical specialty categories as well as clinically relevant care coordination and procedure-based groupings. Some specialties were combined due to overlapping diagnosis domains (eg, “Respiratory and Allergy” and “Orthopedic and Musculoskeletal” domains). The primary focus was for the system grouper diagnoses to be organized around clinical use. For example, “acute myocardial infarction” and “venous thromboembolism” were sorted into the “Cardiovascular and Peripheral Vascular” grouper, while addiction issues, such as “alcohol use disorder,” were sorted into the “Behavioral Health” grouper.

**Table 1.** List of system groupers with example diagnoses.

Condition or specialty grouper	Example diagnosis	Notable deviations
1. Care Coordination	Physical deconditioning, food insecurity, risk for falls	Includes health-related social needs
2. Oncology	Malignancies and radiation therapy diagnoses	Excludes dermatology cancers and includes treatment complications
3. Cardiovascular and Peripheral Vascular	Atrial fibrillation and deep vein thrombosis	Excludes cerebral vascular diagnoses
4. Respiratory and Allergy	Asthma and peanut allergy	—^a^
5. Endocrine	Diabetes mellitus, gout, and hypothyroidism	—
6. Behavioral Health	Schizophrenia and opioid use disorder	—
7. Transplant	Living-related kidney transplant and graft versus host disease	Includes transplant complications
8. Infectious Disease, Immune, or Lymphatic	Pneumonia and immune deficiencies	—
9. Blood	Anemia	—
10. Neurology or Sleep	Seizure and sleep disorders	Excludes chronic pain
11. Ears, Nose, Throat (ENT)	Nasal polyps, cleft palate, and hearing loss	—
12. Fluids, Electrolytes, Nutrition, and Gastrointestinal	Hyponatremia and Crohns disease	—
13. Obstetrics and Gynecology	Ovarian cysts; hemolysis, elevated liver enzymes, low platelet count (HELLP) syndrome; and dense breast tissue	Female-specific diagnoses
14. Genitourinary and Nephrology	Ureteral calculus and prostatitis	Includes male-specific genitourinary issues
15. Dermatology	Atopic dermatitis and melanoma	Includes all dermatology-specific cancers (eg, squamous or basal cell carcinoma)
16. Rheumatology	Rheumatoid arthritis	—
17. Orthopedic and Musculoskeletal	Hip fracture	—
18. Ophthalmology or Eye	Uveitis	Includes complications of eye from other diseases
19. Genetics	Trisomy 21	Includes all nonspecific system genomic issues
20. Pediatrics	28-week prematurity	Includes developmental disorders
21. Surgery, Trauma, Wound, and Pain	Gunshot wound and complex regional pain syndrome	—
22. Other	Edema and medication management	Includes any diagnosis that does not fit into another grouper

a Not applicable.

Due to its polyhierarchal framework, all child-related SNOMED CT concepts include all related downstream concepts, unless excluded by the Boolean logic. In this format, fewer SNOMED CT concepts can represent many derivative *ICD-10* codes more comprehensively than could be achieved by directly curating *ICD-10* codes. For example, 167 SNOMED CT concepts within the Neurology grouper were mapped to 9243 IMO *ICD-10* diagnoses. Our default EHR PLs were reorganized according to this system-based methodology in the order presented in Table 1.

Using our EHR vendor’s built-in tools for grouper build, author EMH iteratively refined the groupers to be consistent with clinical systems using 1211 SNOMED CT concepts. System groupers included the highest parent concept that was appropriate with logic to exclude child-related SNOMED CT concepts not clinically appropriate for a system. For example, squamous cell carcinoma and skin cancers in general are managed clinically by dermatology. In the build for the Oncology grouper, therefore, all dermatologic cancers were excluded and instead added to the Dermatology grouper. As another example, our Cardiovascular grouper includes peripheral vascular diseases like “deep venous thrombosis” but excludes cerebral vascular concepts. This allows diagnoses like “cerebral avascular malformation” to be presented within our Neurology grouper. Both examples highlight the focus of this grouper organization to support clinical specialty coordination of diagnoses.

Multisystem disorders were grouped according to the specialty that would typically manage each disease entity. For example, systemic lupus erythematosus was grouped under “Rheumatology.” If a diagnosis’s SNOMED CT concept was too broad to be captured by one of the 21 groupers, it defaulted into the “Other” category. For example, “edema” is a clinical finding that can be reasonably attributed to multiple diseases. As such, it does not have a specific condition or specialty and instead falls into the “Other” category.

To evaluate the effectiveness of specialty sorting, we used a convenience sample of 50 patients randomly identified in January 2022. These patients had at least 3 encounters in the previous year and were selected across all age groups, ranging from newborn to geriatric patients, with an equal ratio of sexes (Table 2). The encounter criteria ensured that identified patients had multiple recent opportunities to have their PLs updated. The PLs for these patients were extracted through screen capture software by author EMH to develop a cohort with no patient identifiers. Standard EHR PL functionality included system grouper name, time frame since the problem was added to the PL, and a limited free-text overview if included with the entry. These study PL entries were reconfigured into a study document with labels indicating sequential patient number, patient age, and patient sex.

Two of the authors, both family medicine physicians (TT and RS), independently examined each patient’s PL to determine the clinical accuracy of system groupings for all diagnoses (Table 3). For any items whose system attribution they questioned, the reviewers identified the SNOMED CT code attached to the *ICD-10* code. Diagnoses that were deemed correctly grouped into the appropriate system grouper were considered true positives, while those that were incorrectly grouped were considered false positives. All diagnoses in the dropout “Other” category were examined by their associated SNOMED CT code for options for attribution to a defined system grouper. A diagnosis for which the SNOMED CT code was too vague or not specific enough to be grouped was considered a true negative. Any diagnosis that had a SNOMED code that could have been placed in a relevant system grouper but was not was considered a false negative.

**Table 2.** Patient demographics and baseline descriptive statistics. A total of 50 patients, subdivided by age and sex, with descriptive statistics, were reported for each age range.

Age ranges (years)		Gender			Problems			
		Total, n (%)	Male, n (%)	Female, n (%)	Total, n	Mean	Median	Min-Max
<1		6 (12)	3 (50)	3 (50)	72	12.0	10	6-20
1-17		7 (14)	3 (42.9)	4 (57.1)	157	22.4	26	4-35
18-64		24 (48)	11 (45.8)	13 (54.2)	342	14.3	10	4-43
≥65		13 (26)	8 (61.5)	5 (38.5)	298	22.9	21	4-59
All ages		50 (100)	25 (50)	25 (50)	869	17.4	12	4-59

**Table 3.** Description of metrics used to determine the effectiveness of automated system grouping. Two reviewers examined individual problem list items and their assigned grouping, placing each into a category.

Assessment category	Definition	Example
True positive (correct system association)	Diagnosis falls into the right disease system—the SNOMED^a^ grouper is specific and attributable.	“Community-acquired pneumonia” in the Infectious Disease system
False positive (incorrect system association)	Diagnosis falls in the wrong system grouper.	“Diaphragmatic stimulation by cardiac pacemaker” grouped under “Central Hypoventilation Syndrome”.
True negative (Other—correct system association)	The SNOMED grouper associated with a diagnosis is not specific enough to be in anything but the Other category.	“Anticoagulated” placed with the SNOMED grouper “Drug therapy finding”. This is not specific enough to be attributed to just anticoagulation status.
False negative (Other—incorrect system association)	Diagnosis belongs to a specified system grouper but falls into the Other category due to logic deficits in the grouper.	“Genetic disorder” falling into the “Other” category until the VCG Grouper is corrected.

a SNOMED: Systematized Nomenclature of Medicine.

Each diagnosis was independently categorized according to the scheme in Table 3; then the reviewers compared their determinations. A third independent clinician (author LN) served as a tie-breaker for those PL items for which an agreement was not reached. We calculated descriptive statistics to summarize the volume of diagnoses for the 50 test patients and performance metrics to assess the accuracy and validity of the groupers. The correlation coefficient (κ statistic) was calculated for the degree of agreement between reviewers and for SNOMED CT grouper attributions.

This work was performed using Epic Systems (version May 2021; Verona, WI) initially deployed with ambulatory applications in July 2012 and inpatient applications in June 2013 within the Duke University Health System.

### Ethical Considerations

All patient data were anonymized with all demographic identifiers removed except for age. This study was approved by the Duke University Internal Review Board for exempt status (IRB #PRO-00108903).

## Results

Across the 50 patients, aged 14 days to 93 years, there were a total of 869 (range 4-59) diagnoses identified, with a median of 12 diagnoses per patient. Table 2 includes the breakdown of the volume of PL entries across age and sex.

After their independent evaluations of the PLs, the reviewers initially agreed on 821 (94.4%) of the 869 total problems (Cohen κ coefficient of 0.7, indicating moderate agreement [21]). Of the remaining 48 diagnoses, they subsequently agreed on 32 for a revised agreement rate of 98.2%. The remaining 16 were adjudicated by author LN for attribution.

**Figure 2.**
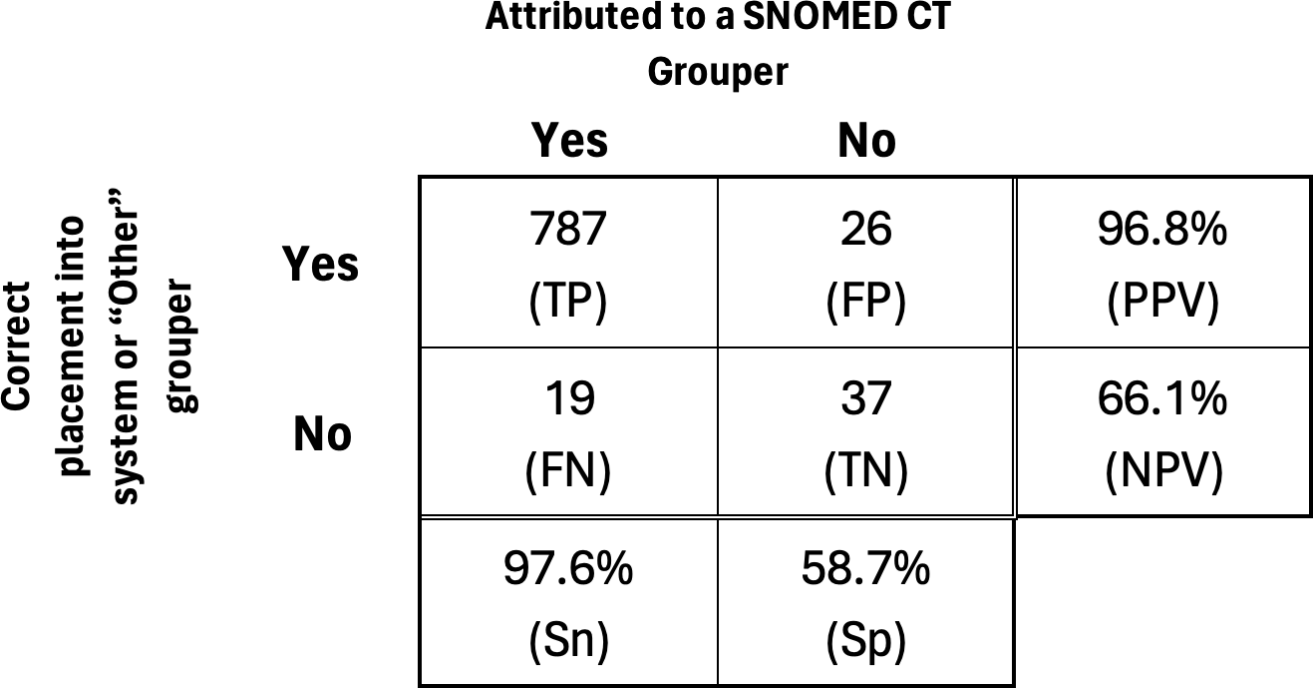
Two clinicians’ review of problem list sorting algorithm. FN: false negative; FP: false positive; PPV: positive predictive value; NPV: negative predictive value; TN: true negative; TP: true positive; Sn: sensitivity; SNOMED CT: Systematized Nomenclature of Medicine Clinical Terms; Sp: specificity.

Based on the definitions presented in Table 3, Figure 2 describes our results. Our final attribution evaluation found that the diagnoses were correctly attributed to a system grouper (ie, sensitivity) in 97.6% of cases, and the nonspecific diagnoses were correctly placed in the “Other” category (ie, specificity) in 58.7% of cases. The positive predictive value, or the correct grouper accuracy rate, was 96.8%. We found 37 (4.3%) true negatives, representing concepts without a SNOMED CT code or diagnoses too general to be attributed to a clinical system. The calculated *F*_1_-Score was 0.972.

## Discussion

### Overview

The PL is the repository of medical diagnoses intended to reflect the patient’s clinical conditions. Without groupings reflective of the larger specialty formats of clinical care, the PL can become overloaded and difficult to use as a tool to communicate a patient’s clinical status across encounters. We developed 21 SNOMED CT groupers for system concepts to standardize the organization of our EHR PL based on 1211 concepts (Table 1). We chose to evaluate these PL groupers across all ages and sexes to provide a more representative sample of diagnoses across our EHR patient population, recognizing that this is only a subset of the total potential diagnoses. Taking advantage of the hierarchal logic of the SNOMED CT concepts refined with Boolean logic allowed for more than 95% of the diagnoses to be attributed to a system grouper [22,23].

We established the effectiveness of these SNOMED CT groupers in organizing the PL by clinical system related to clinical specialty or condition. We propose that this standardized format for PL organization permits the sharing and reproduction of concepts across other health systems and EHRs.

### Comparison to Prior Work

Other groups have used conceptually similar methods with SNOMED CT codes for clinical phenotyping [22]. However, those code sets are typically more narrow in scope for a more specific clinical description. The United Medical Language System Clinical Observations Recordings and Encoding Problem List Subset is meant to “facilitate the use of SNOMED CT as the primary coding terminology for PLs or other summary level clinical documentation” [24]. Compared to these other SNOMED CT code sets, our implementation includes broader clinical coordination groupings (eg, surgical, transplant, care coordination, and infectious disease) that are more reflective of the PL clinical care needs within our institution. Our work here builds upon those efforts and applies them at the system level, which is more accessible for clinical use.

### Limitations

We noted some limitations and challenges in using SNOMED CT concepts for this build. Despite ongoing international mapping efforts [17,18], SNOMED CT concepts are not fully representative of all the *ICD-10* codes because of differences in original intended uses [8,13] and baseline granularity [25,26]. For example, the *ICD-10* code “Encounter for pre-transplant evaluation for chronic liver disease” is mapped to the SNOMED CT concept “patient encounter status,” as there is no other comparable SNOMED CT coding option. Estimates for the proportions of completely mapped concepts or codes are found in studies reviewing the automation of mapping SNOMED CT and *ICD-10* codes; one study estimated the proportion of complete mappings to *ICD-10-Clinical Modification* (*ICD-10-CM*) at 74% in 2012 [25], and another one estimated the proportion of complete mappings to *ICD-10-Procedure Coding System* (*ICD-10-PCS*) (used to capture inpatient procedures) to be about 86% in 2017 [27].

There were many *ICD-10* diagnoses that were too broad to easily match a SNOMED CT system grouper. “Fatigue” is a good example of an inherently vague constitutional or multisystemic symptom that does not have a clearly identifiable system-level grouper in our schema. For these diagnoses, the “Other” category was used to capture the remaining nonspecific diagnoses. It is important to note that this category is not the same as the *ICD-10* options for “Not Otherwise Specified“ (NOS) or “Not Elsewhere Classifiable” (NEC) codes for lesser defined diagnoses. For example, “Pneumonia due to other infectious organisms, NEC” still falls into our “Infectious Disease, Immune, Lymphatic” grouper.

We also note that the mappings are not completely represented across all specialties in terms of the breadth of coverage of concepts. For example, we found more SNOMED CT cardiology-specific concepts and fewer pediatric-specific concepts. These differences may reflect the relative volume of cardiology diagnoses in the general population. The more specific diagnosis of “Encounter for assessment of implantable cardioverter-defibrillator” was mapped to an appropriate SNOMED CT concept and was correctly placed into our cardiovascular system grouper. However, the pediatric diagnosis “Concern about growth” was only mapped to the SNOMED CT code “Finding reported by subject or history provider,” which was too broad to be added to the Pediatric grouper only, consequently falling into the “Other” category. Specialties such as pediatrics also require greater levels of specificity for their diagnoses than is always possible with the SNOMED CT concepts currently available.

There were also multiple *ICD-10* codes mapped to the same SNOMED CT code that made attribution to a system grouper challenging. For example, the diagnoses “Diaphragmatic stimulation by pacemaker” and “Disorder of cardiac pacemaker system” mapped to the same SNOMED CT code of “Disorder of cardiac pacemaker system,” placing them into the Cardiovascular grouper, although the former would ideally be attributed to the Pulmonary grouper.

As we consider the future challenges of algorithm-based PL sorting, it will be important to investigate the implications of updating ontologies as the World Health Organization has already published the 11th edition of *ICD* (*ICD-11)* with 35 countries now implementing it [28]. We do not suspect that *ICD-11* will replace SNOMED CT as an ontology organization method, as SNOMED CT maintains greater flexibility for clinical use. Health systems are always evolving, and it will be important to consider how such algorithms and their applications will evolve within them.

### Conclusions

We leveraged a PL sorting algorithm based on the clinical system–based SNOMED CT groupers to create a standardized PL format in our EHR, reorganizing the diagnoses, symptoms, and medical problems for better clinical utility. We found subjective positive outcomes for our clinical users who reported streamlining their clinical review processes and easier ability to identify similar and duplicate diagnoses. This may be especially helpful for patients with complex issues and many associated diagnoses. A structured PL also enables a shift from patient-level evaluation to potentially population-level assessments and cleanup automation.

As with improvements in the provider experience, automated PL maintenance may also impact researchers leveraging PL diagnoses for machine learning and other similar research. Such possibilities underscore the need for accurate and updated PL diagnoses to achieve and maintain high-fidelity outputs. It will be important to further evaluate methods to automate the maintenance of accurate Pls and best influence care delivery.

## Abbreviations

EHR: electronic health record
HITECH: Health Information Technology Economic and Clinical Health
*ICD*: *International Classification of Diseases*
IHTSDO: International Health Terminology Standards Development Organization
IMO: Intelligent Medical Objects
NEC: Not Elsewhere Classifiable
NOS: Not Otherwise Specified
PL: problem list
SNOMED: Systematized Nomenclature of Medicine
SNOMED CT: Systematized Nomenclature of Medicine Clinical Terms
SOAP: Subjective, Objective, Assessment, and Plan
